# Interventions to promote social connection and their effect on depression: An umbrella review

**DOI:** 10.1192/j.eurpsy.2024.482

**Published:** 2024-08-27

**Authors:** L. De Risio, M. Pettorruso, A. D’Onofrio, M. C. Vicinelli, C. De Troia, M. Santorelli, M. Boffa, P. Politi, G. Martinotti, F. Zoratto, M. Borgi

**Affiliations:** ^1^Department of Psychiatry and Addiction, ASL Roma 5, Colleferro; ^2^Department of Neuroscience, Imaging and Clinical Sciences, G. d’Annunzio University of Chieti-Pescara , Chieti; ^3^Center for Behavioral Sciences and Mental Health, Istituto Superiore di Sanità, Rome; ^4^Department of Brain and Behavioral Sciences, University of Pavia, Pavia, Italy

## Abstract

**Introduction:**

Social connection (SC) is a multi-dimensional concept capturing both the structural–quantitative (e.g., number of social relations, social contact frequency, network structure) and the functional–qualitative dimension (e.g., social support) of social relationships. Although empirical evidence of the association between SC measures and depression has increased significantly in recent years (De Risio et al, *J Affect Disord* 2024; 345 358–368), very little is known about the extent to which interventions that build SC are effective in improving depressive symptoms.

**Objectives:**

This umbrella review of systematic reviews/meta-analyses aims to synthesize evidence regarding the effectiveness of SC interventions on depression. Our primary focus is on interventions directly acting upon the natural social network, while indirect interventions that aim to improve social skills, or those that provide professional (formal) or semi-professional support through health services, were excluded.

**Methods:**

We provide a synthesis of the consistency and magnitude of the effectiveness of SC interventions on depression. We searched PubMed, PsycINFO, Cochrane Library, and EMBASE and 16 reviews/meta-analyses were included. Information on the effectiveness of SC interventions on depression were compared among different populations. The quality/certainty of evidence was assessed using AMSTAR-2 and GRADE tools.

**Results:**

Included interventions were categorized into the following domains: social support (interventions increasing both perceived and enacted social support from family, friends, and others); social engagement (interventions aimed at strengthening social networks and contrasting social isolation); social inclusion (interventions promoting social integration and access to social capital); social identification (interventions enhancing participants’ identification with a group). Overall, the evidence is rather mixed with some SC interventions resulting in little to no difference in depressive symptoms compared to usual care/other interventions. The most promising interventions appear to be those contrasting social disengagement and reducing social isolation in older individuals and in patients with depression, as well as social inclusion interventions for adolescents and young adults.

**Conclusions:**

The broader implications of SC as a key determinant of depression call for a deep examination of the impact of interventions/preventive programs on the evolving psychopathology of depressive trajectories and inform on which targeted interventions are more effective, thus guiding public health policies.

**Disclosure of Interest:**

None Declared

